# An improved YOLOv8n model for in-field detection of pests and diseases in pakchoi

**DOI:** 10.3389/fpls.2025.1730683

**Published:** 2026-01-22

**Authors:** Yi Zhu, Yanlu Han, Yilu Yin, Shuo Zhao, Yubin Lan, Danfeng Huang

**Affiliations:** 1 College of Agricultural Engineering and Food Science, Shandong University of Technology, Zibo, China; 2 Institute of Modern Agricultural Equipment, Shandong University of Technology, Zibo, China; 3 Zibo Digital Agriculture and Rural Development Center, Zibo, China; 4 School of Agriculture and Biology, Shanghai Jiao Tong University, Shanghai, China

**Keywords:** loss function, object detection, pakchoi, pest and disease recognition, YOLO model

## Abstract

As an important leafy vegetable, pakchoi (*Brassica chinensis* L.) frequently suffers from pests and diseases in field environments. These symptoms are often localized on specific leaf regions, resulting in substantial losses in yield and quality. To achieve efficient and accurate detection of pakchoi pests and diseases, this study proposes an improved lightweight object detection model, termed YOLOv8n-DBW, based on the YOLOv8n framework. First, the original C2f module in the backbone network is replaced with a novel C2f-PE module, which integrates Partial Convolution (PConv) and an Efficient Multi-Scale Attention (EMA) mechanism to enhance high-level semantic feature extraction and multi-scale information fusion. Second, a Weighted Bidirectional Feature Pyramid Network (BiFPN) is introduced into the neck network to strengthen multi-scale feature fusion while improving model generalization and lightweight performance. Finally, the original CIoU loss in the regression branch is replaced with the Wise-IoU (Weighted Interpolation of Sequential Evidence for Intersection over Union) bounding box loss function, which improves bounding box regression accuracy and significantly enhances the detection of small and irregular pest and disease targets. Experimental results on a field-collected pakchoi pest and disease dataset demonstrate that the proposed YOLOv8n-DBW model reduces the number of parameters and model size by 33.3% and 31.8%, respectively, while improving precision and mean average precision (mAP) by 5.0% and 7.5% compared with the baseline YOLOv8n model. Overall, the proposed method outperforms several mainstream object detection algorithms and provides an efficient and accurate solution for real-time pakchoi pest and disease detection, showing strong potential for deployment on embedded systems and mobile devices.

## 1 Introduction

Pakchoi (*Brassica chinensis* L.) is a leafy vegetable of significant economic and nutritional importance in Asia, particularly in China. It is widely cultivated due to high consumer demand (Wu et al., 2025). In 2022, the cultivation area of pakchoi in China reached approximately 300,000 hectares, yielding a total production of around 18 million tonnes, thereby making a substantial contribution to the stability of the vegetable supply. However, the continuous expansion of cultivation and increasing diversification of varieties have intensified the challenges posed by pests and diseases (Hou et al., 2018). Frequent occurrences of pests and diseases—including the Diamondback Moth (Levere and Bresnahan, 2024), Downy Mildew (Liu et al., 2024), Leaf Miner (Vilela et al., 2023), Alternaria Leaf Spot (Olmez et al., 2023), Black Rot (Kellner et al., 2022), White Rust (Awika et al., 2019), and White Spot (Mamede et al., 2022)—severely threaten the efficiency and sustainability of pakchoi production. Consequently, the rapid and accurate identification of these pests and diseases (Zhang et al., 2024), followed by the formulation and implementation of precise integrated management strategies, has become a critical scientific challenge and an urgent priority in agricultural research.

In the research field of pakchoi pest and disease recognition, traditional identification approaches have long been heavily reliant on manual identification, which not only requires substantial human resources, but also suffers from inconsistent recognition results and low overall accuracy. To address these limitations and substantially improve recognition precision and efficiency, early-stage automated research primarily explored digital image processing techniques (Song et al., 2022) and conventional machine learning algorithms (Liu, 2022). However, models constructed through such methods predominantly depended on manually engineered features and architectures, resulting in severely constrained flexibility and scalability that hindered their adaptation to complex and dynamic real-world scenarios. Globally, substantial research achievements have been accumulated in the field of crop pest and disease recognition. Driven by the rapid advancements in artificial intelligence, the applications of deep learning have gradually expanded from the early-stage identification of characteristic diseases in single crops to complex scenarios involving multiple crops and pathogen types (Ai et al., 2020). For instance, the implementation of diverse deep learning models in crops such as maize (Chenrui et al., 2022), tomato (Saeed et al., 2023), wheat (Genaev et al., 2021), and cotton (Caldeira et al., 2021) has provided robust technical support for the precision prevention and control of pests and diseases during crop cultivation (Mu and Zeng, 2019; Xin and Wang, 2021; Wang, 2022).

In recent years, detection methods based on the YOLO deep learning model have been increasingly applied due to their high speed and accuracy. For instance, to address the insufficient feature extraction efficiency of the original YOLOv5l model in cucumber pest and disease detection, researchers replaced the C3 modules in both the backbone and neck with Bottleneck CSP modules, constructing a more efficient feature learning pathway. The improved model achieved a mean average precision (mAP) of 80.1% (Omer et al., 2024). In tomato pest and disease recognition, an improved YOLO-FMDI deep learning algorithm demonstrated significantly enhanced accuracy compared to the original YOLOv8n (Sun et al., 2024). To tackle core issues in vegetable disease detection, such as missed detection of small targets, insufficient feature fusion, and imbalance between detection accuracy and speed, the YOLOv8n-vegetable improved model was proposed. This model achieved an mAP of 91.4%, a 6.46% improvement over the original YOLOv8n (Wang and Liu, 2024). Similarly, Zheng et al. (2024) proposed the YOLOPC model based on YOLOv5s for pakchoi pest detection, achieving an mAP of 91.4%, representing a 12.9% increase over the original YOLOv5s. These aforementioned studies have carried out intelligent recognition of vegetable pests and diseases, successfully achieving precise identification of various pests and diseases. Despite certain progress in the research on the intelligent recognition of pests and diseases, some limitations still exist. Given the characteristic differences between different crops and pests/diseases, it is necessary to design recognition models in a targeted manner to achieve precise identification. Moreover, in the face of pests and diseases with a wide variety of species and variable symptoms, existing recognition methods face challenges in practical application. For example, detection results are susceptible to environmental factors, and both recognition efficiency and accuracy need to be improved. Additionally, research on pest and disease recognition models specifically for leafy vegetables is still relatively scarce at present.

Addressing the aforementioned issues and leveraging the outstanding performance of the YOLO series networks in object detection—particularly the advantages of YOLOv8 in detection accuracy, speed, and model size (Wang et al., 2023). This present work constructs the YOLOv8-DBW model for pest and disease detection in pakchoi, building upon the framework of YOLOv8n. Furthermore, the YOLOv8-DBW model is compared with classical object detection models, namely SSD (Zhai et al., 2020), Faster R-CNN (Zhao and Liu, 2024), YOLOv5n (Ma et al., 2023), YOLOv5s(Xie et al., 2024), YOLOv7-tiny (Cheng et al., 2024), YOLOv10n (Li et al., 2024), YOLOv11n (Zhou and Jiang, 2025), and YOLOv12n (Yin et al., 2025), to evaluate the efficiency and accuracy. The proposed model not only significantly enhances detection performance for pests and diseases but also offers a reliable technical solution for lightweight, real-time diagnosis in complex field conditions. These advancements holds substantial practical implications for promoting the intelligent development of precision agriculture. The research findings can provide efficient and accurate technical support for pakchoi pest and disease detection.

## 2 Materials and methods

### 2.1 Data collection

**Figure 1:**
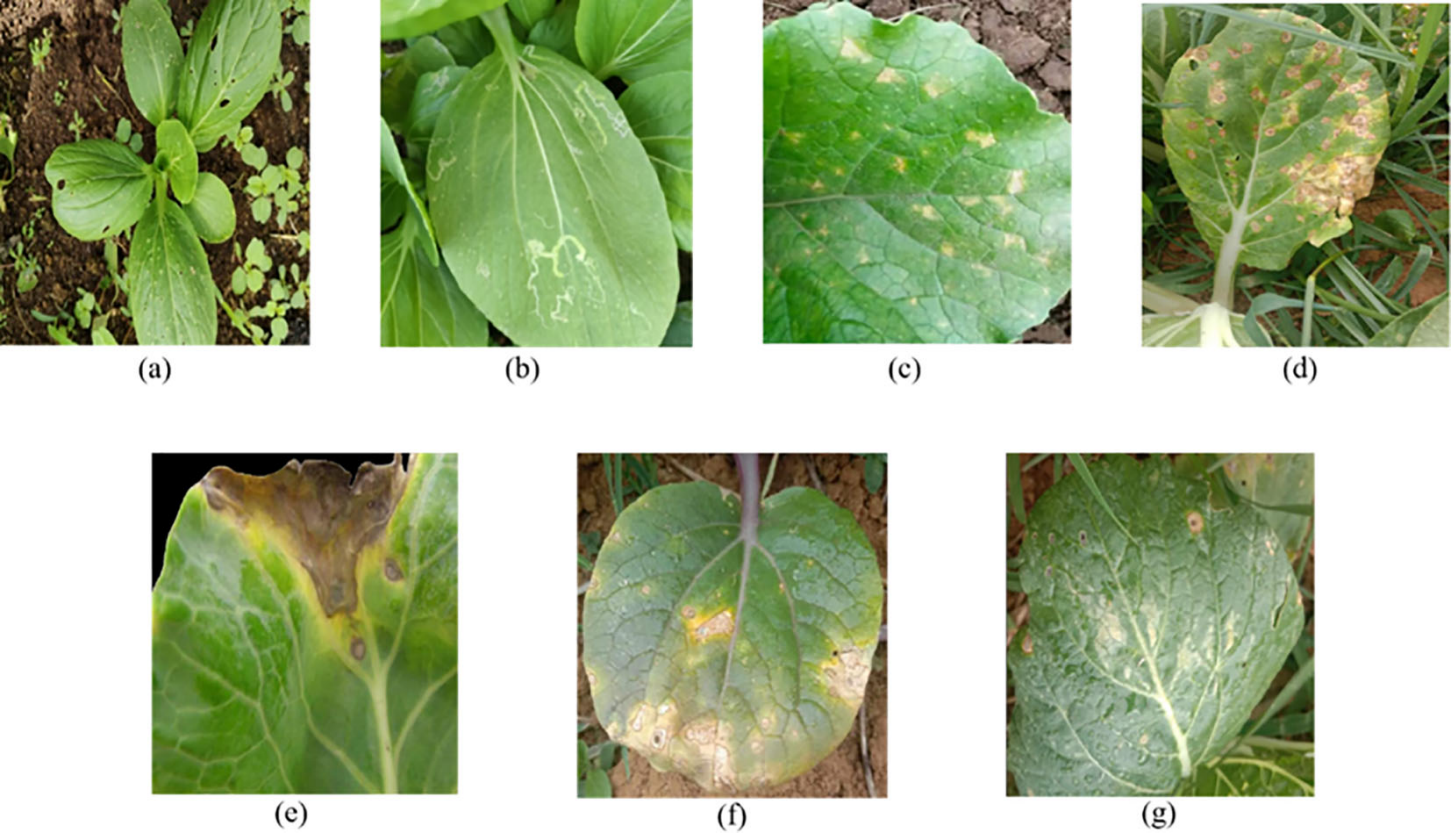
Image examples of the data set. **(a)** is the Diamondback Moth, **(b)** is the Leaf Miner, **(c)** is the Downy Mildew, **(d)** is the Alternaria Leaf Spot, **(e)** is the Black Rot, **(f)** is the White Rust, **(g)** is the White Spot.

Seven types of pakchoi pests and diseases that frequently occur in production were selected as research targets: Diamondback moth, Leaf Miner, Downy Mildew, Black Spot, Black Rot, White Rust, and White Spot. Detailed visual characteristics of these pests and diseases are summarized in **Table 1**, and representative image samples are shown in **Figure 1**. To minimize selection bias and ensure a representative coverage of different infection stages, a systematic scanning protocol was adopted during image acquisition. Images of pakchoi plants were captured sequentially along cultivation rows to include early-stage, mild, atypical, and late-stage symptomatic samples. Shooting distances were standardized between 20 and 50 cm to balance feature resolution and field of view. Images were primarily captured from a vertical top-down perspective, with additional 45° oblique views to account for leaf overlap and variations in plant morphology. Image acquisition was conducted from March 21 to May 8, 2025, in Cao County (Shandong Province), Wujiang District (Jiangsu Province), and Jiading District (Shanghai). Images were collected using multiple commonly used smartphone models, including the Xiaomi 12X, OPPO Reno8 Pro, and Samsung Galaxy A53. The corresponding shooting resolutions were 4000×3000, 3024×4032, and 5632×4224 pixels, respectively.

**Table 1:** The common diseases and pests of pakchoi and key characteristics.

Names of pests and diseases	Feature description
Downy Mildew	On the leaf adaxial surface (upper side), irregular chlorotic to yellowish-brown lesions appear. Under humid conditions, a downy mildew growth (white or grayish-purple in color) develops on the corresponding areas of the leaf abaxial surface (lower side). As the lesions expand, the affected leaves become chlorotic, yellow, curled, and withered.
Alternaria Leaf Spot	Circular or subcircular, brown to dark brown lesions appear on the leaves, often exhibiting distinct concentric rings. As the disease progresses severely, lesions coalesce and expand, ultimately causing leaf necrosis.
Black Rot	V-shaped yellowish-brown lesions initially appear at the leaf margins and progressively expand inward, with characteristic vein blackening forming a net-like pattern at lesion boundaries.
White Rust	On the abaxial leaf surface, cream-colored, slightly raised pustules (sori) develop, which rupture to release powdery white spores. Correspondingly, the adaxial surface exhibits indistinct chlorotic spots ranging from pale yellow to yellowish-green. As the disease progresses, lesions coalesce, leading to extensive chlorosis and necrosis of the foliage.
White Spot	Initial lesions manifest as small smoky grey-brown specks that subsequently undergo radial expansion, forming circular to subcircular dichromatic lesions characterized by ash-white centers surrounded by distinct lemon-yellow margins. Under humid conditions, the abaxial surface corresponding to these lesions develops effuse, pale grey fungal growth.
Leaf Miner	Larvae tunnel endophagously through the mesophyll tissue, forming characteristic serpentine mines exhibiting a whitish, serpentine trajectory through the foliar layers.
Diamondback Moth	Larvae feed on the mesophyll, early-instar larvae leave translucent feeding spots, while late-instar larvae bore holes; in severe infestations, only the vascular veins remain.

### 2.2 Data enhancement

**Figure 2:**
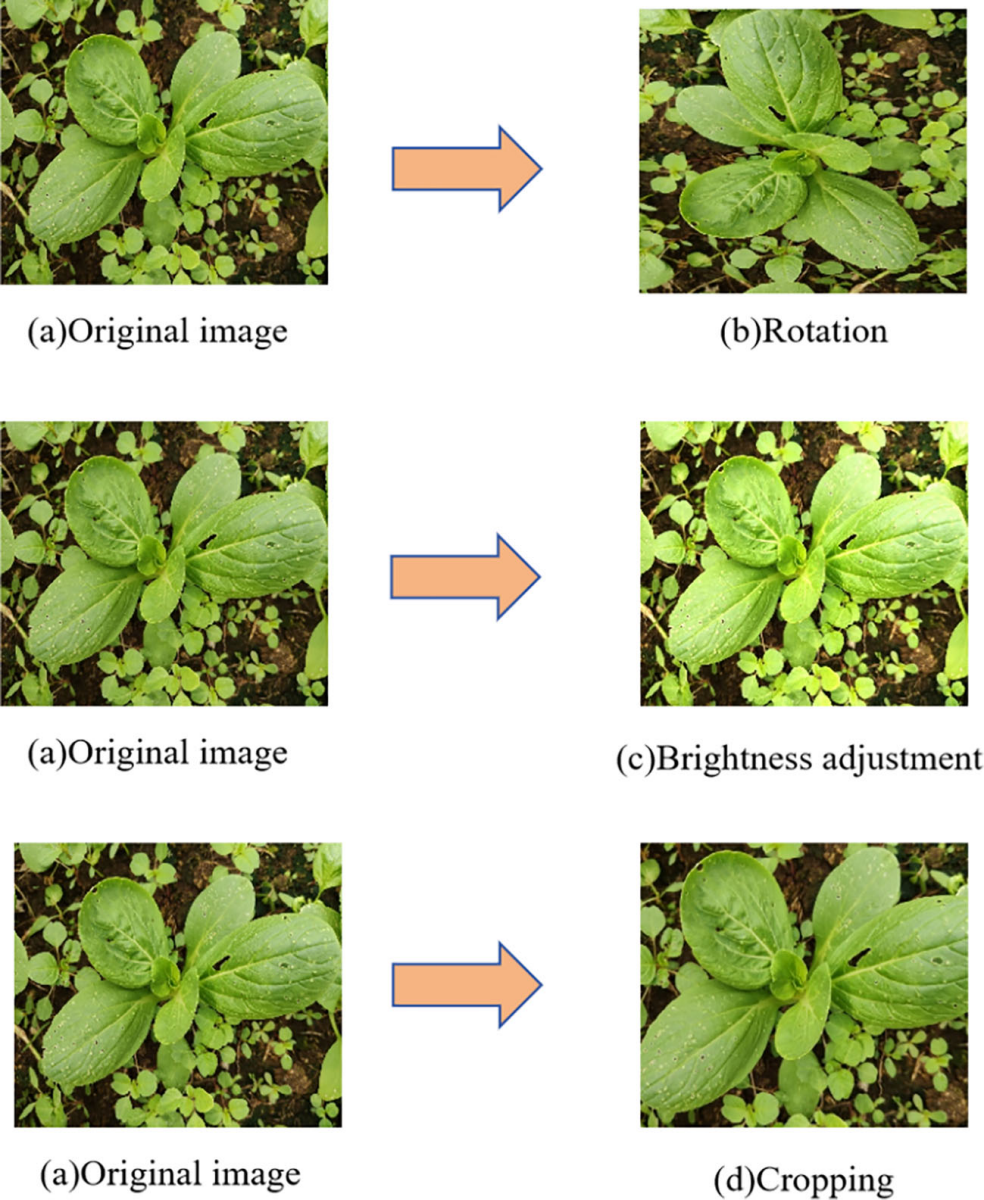
Data enhancement.

In this study, we collected images of seven common pests and diseases of pakchoi from three different field areas, covering various growth stages and disease manifestations. Given the significant variability in illumination and meteorological conditions in open-field environments, the data acquisition process was designed to encompass multiple diurnal phases (morning, noon, afternoon) and diverse weather scenarios (sunny, overcast, post-precipitation periods). The original dataset consisted of 1,782 images. To enhance the model’s generalization ability, we performed data augmentation on these images (as shown in **Figure 2**), resulting in a final dataset of 6,110 images. The sample distribution across the seven pest and disease categories is as follows: 1,085 images of Diamondback Moth damage; 842 images of Leaf Miner disease; 992 images of Downy Mildew disease; 753 images of Alternaria Leaf Spot disease; 745 images of Black Rot disease; 855 images of White Rust disease; and 838 images of White Spot disease. All images in the final dataset were standardized to a resolution of 640×640 pixels in JPG format. Furthermore, the dataset included images captured during “post-precipitation” periods, which naturally contained samples with water droplet reflections and soil splashes. Meanwhile, variations in handheld movement during image capture introduced natural motion blur effects, ensuring the model’s robustness against complex field challenges.

### 2.3 Data labeling

The dataset images were manually annotated using LabelImg software (https://github.com/HumanSignal/labelImg). The following categorical labels were assigned: “Backmoth” for Diamondback Moth, “Leafminer” for Leaf Miner, “Mildew” for Downy Mildew, “ALTERNARIA” for Alternaria, “BLACK-ROT” for Black Rot, “WHITE-RUST” for White Rust, “WHITE-SPOT” for White Spot. All annotations were saved in TXT files, each containing the corresponding object class and bounding box coordinates. Multi-instance annotations were preserved where applicable, with individual images containing simultaneous occurrences of multiple pathologies. The dataset was subsequently partitioned into training, validation, and test sets with an 8:1:1 ratio, resulting in 4,888 images for training, 611 for validation, and 611 for testing.

### 2.4 The network structure of the YOLOv8 deep-learning model

**Figure 3:**
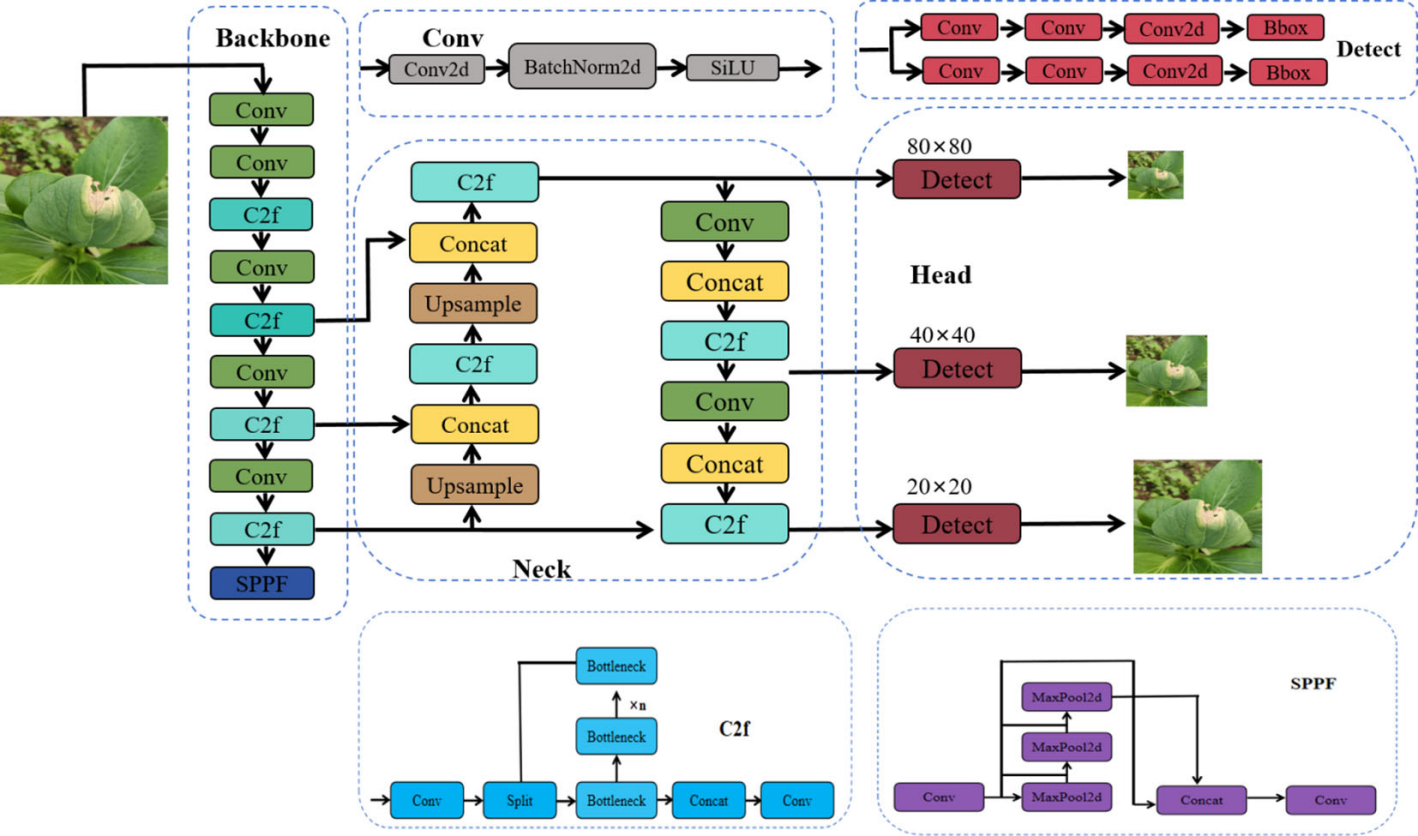
YOLOv8 model network structure. Conv is the convolution module, C2f is the cross-stage partial feature fusion module, SPPF is the spatial pyramid pooling layer, Concat is the feature concatenation module, Upsample is the upsampling layer, Detect is the detection head, Conv2d is the two-dimensional convolution, BatchNorm2d is the batch normalization layer, SiLU is the activation function, MaxPool2d is the max pooling layer, Bottleneck is the convolution module that includes a residual connection, n denotes the number of Bottleneck modules, Split as the feature hierarchization, Bbox refers to the bounding box.

As a next-generation end-to-end object detection algorithm, YOLOv8 significantly enhances detection performance in complex scenarios through architectural refinements and technical innovations (Ma et al., 2024), while inheriting the computational efficiency characteristic of the YOLO series. The model employs a four-module architecture: Input → Backbone → Neck → Head (**Figure 3**), and its core design demonstrates in-depth optimizations for real-time performance, adaptability to multi-scale targets, and model lightweighting. The model has five scaled versions (n, s, m, l, x), which satisfy the adaptation requirements of diversified application scenarios (Liu et al., 2023; Ma and Pang, 2023; Wang et al., 2023; Chen et al., 2025; Long and Lin, 2025).

This study adopts YOLOv8 as the baseline network model, following the canonical “Backbone-Neck-Head” hierarchical design paradigm, and is collaboratively composed of three core functional modules to form an efficient object detection framework (Huang et al., 2024). Among these, the backbone network, serving as the primary structure for feature extraction, incorporates the Basic Convolution (Conv) module, the Cross-Stage Partial Feature Fusion (C2F) unit, and the Spatial Pyramid Pooling Fast (SPPF) module (Xiao et al., 2024). The neck network adopts a bidirectional architecture that integrates the Feature Pyramid Network (FPN) (Xie et al., 2023) and the Path Aggregation Network (PAN) (Roy et al., 2022). Via a bidirectional connection mechanism involving top-down semantic feature transmission and bottom-up detailed feature feedback, it realizes cross-scale fusion of feature maps across different levels. The detection head employs an Anchor-Free detection approach, doing away with the reliance of traditional anchor-based mechanisms on prior target sizes (Wang et al., 2023).

However, during training on the pakchoi pest/disease dataset, the original YOLOv8 model exhibited insufficient detection accuracy and a low target recognition rate (Yue et al., 2024). To evaluate the performance differences among various model versions, comparative experiments were conducted on the YOLOv8 series (n/s/m/l/x). Mean Average Precision (mAP) served as the core evaluation metric to assess the detection performance variations across models under conditions of multi-scale target distribution and leaf occlusion scenarios. The results are detailed in **Table 2**.

**Table 2:** Performance results of the YOLOv8 series version.

Model	Mean average precision @0.5(mAP@0.5)/%	Mean average precision @0.5:0.95(mAP@0.5:0.95)/%	Parameters/M
YOLOv8n	77.8	59.5	3.0
YOLOv8s	75.4	58.9	11.1
YOLOv8m	73.8	57.7	25.8
YOLOv8l	77.6	60.6	43.6
YOLOv8x	78.9	61.9	68.1

mAP@0.5 represents the average accuracy at a recall rate equal to 0.5.mAP@0.5:0.95 indicates average accuracy in the 0.5 to 0.95 recall range.

As shown in **Table 2**, under the unified training configuration (200 epochs, RTX 4090 GPU, and identical hyperparameters), despite their larger parameter counts and more complex architectures, the YOLOv8m and YOLOv8l models exhibited lower detection accuracy on the pakchoi pest/disease dataset compared to the lightweight YOLOv8n model. This indicates that merely increasing model complexity failed to yield accuracy gains on this specific dataset, while significantly increasing the computational burden and inference time. Although YOLOv8x achieved the highest accuracy, its substantial parameter count resulted in excessively slow inference speeds, rendering it impractical for low-cost, high-efficiency real-world applications. In addition, the YOLOv8s model also demonstrated slightly lower accuracy than YOLOv8n. This phenomenon may be attributed to the fact that larger models with higher parameter counts typically require larger datasets or different convergence schedules to avoid redundancy and potential overfitting in specific agricultural scenarios. Considering the balance between detection accuracy, computational cost, and inference speed, YOLOv8n was selected as the baseline model for subsequent improvements. This model maintains relatively high detection accuracy while possessing the lowest parameter count and highest inference efficiency, serving as a solid foundation for algorithmic optimization.

### 2.5 Improved YOLOv8 model network structure

**Figure 4:**
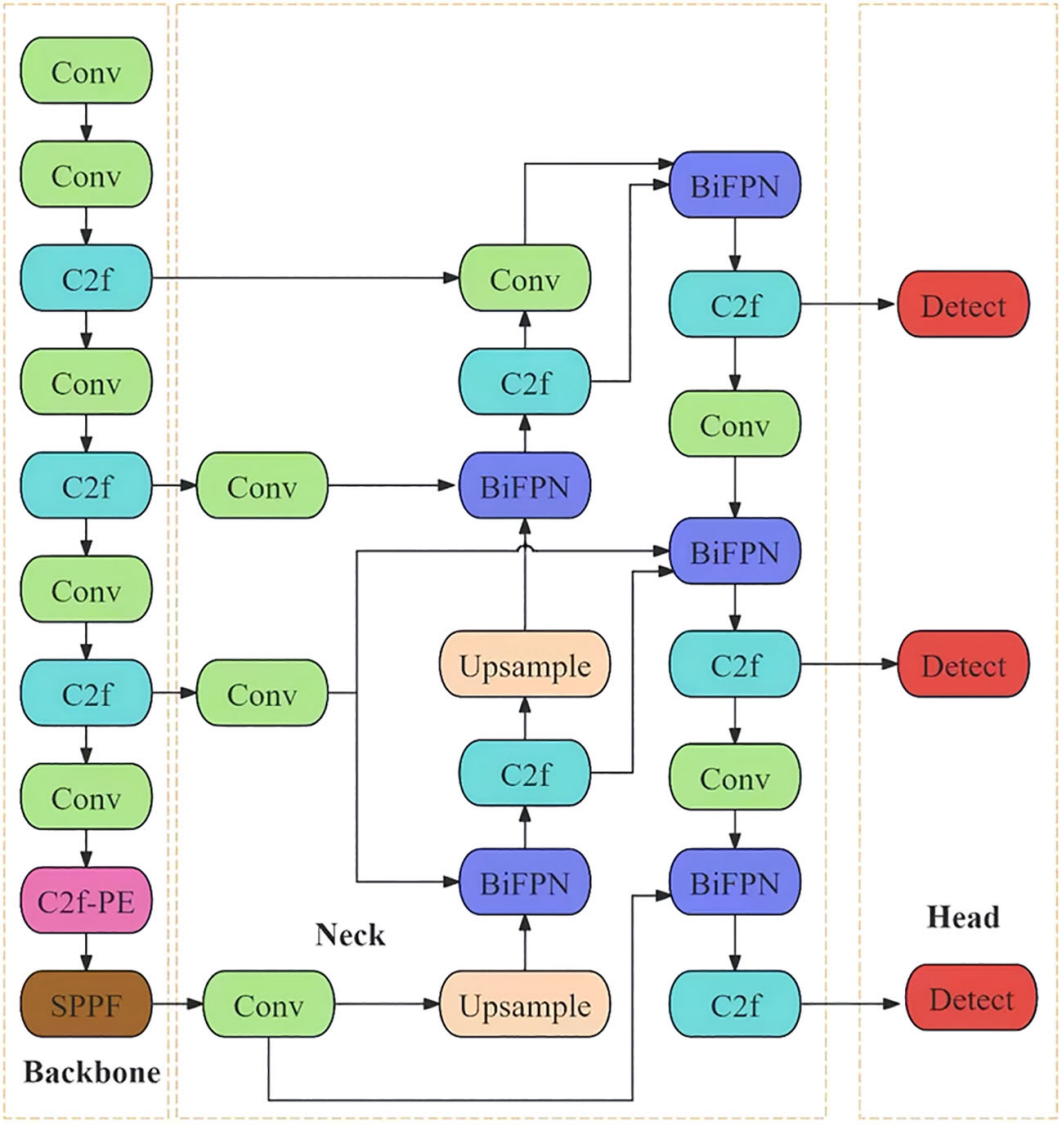
Network structure of the improved YOLOv8n model.

In natural settings, pakchoi exhibits high-density planting, leading to challenges such as mutual leaf occlusion, weed interference, and overlapping leaves across different growth stages. Concurrently, pest and disease regions display high diversity in characteristics: pathogen infection manifests as lesions with distinct textures, morphologies, and colors, while insect damage presents as mechanical injuries like mines and holes. This complex background interference coupled with significant morphological variations in pests and diseases complicates the precise identification and localization of target regions by detection models. To address these challenges, this study proposes the YOLOv8-DBW model, with the improvement strategy comprising the following three key aspects:

1. Backbone Network Enhancement: An efficient multi-scale attention mechanism and partial convolution structure are introduced to enhance the model’s ability to extract small-target features in complex field environments, thereby improving recognition accuracy.
2. Neck Network Enhancement: The BiFPN module is introduced to strengthen feature fusion capability while notably reducing model parameter count and computational cost, thus achieving lightweight design.
3. Loss Function Optimization: The Wise-IoU (Weighted Interpolation of Sequential Evidence for Intersection over Union) loss function is introduced, which incorporates classification information into the Intersection over Union (IoU) computation to enhance the model’s bounding box regression performance. The refinement improves learning precision for pest/disease features, thereby boosting detection stability and accuracy. The architecture of the enhanced YOLOv8-DBW model is illustrated in **Figure 4**.

#### 2.5.1 C2f-PE module integrating efficient multi-scale attention and partial convolution

##### 2.5.1.1 Efficient multi-scale attention mechanism

**Figure 5:**
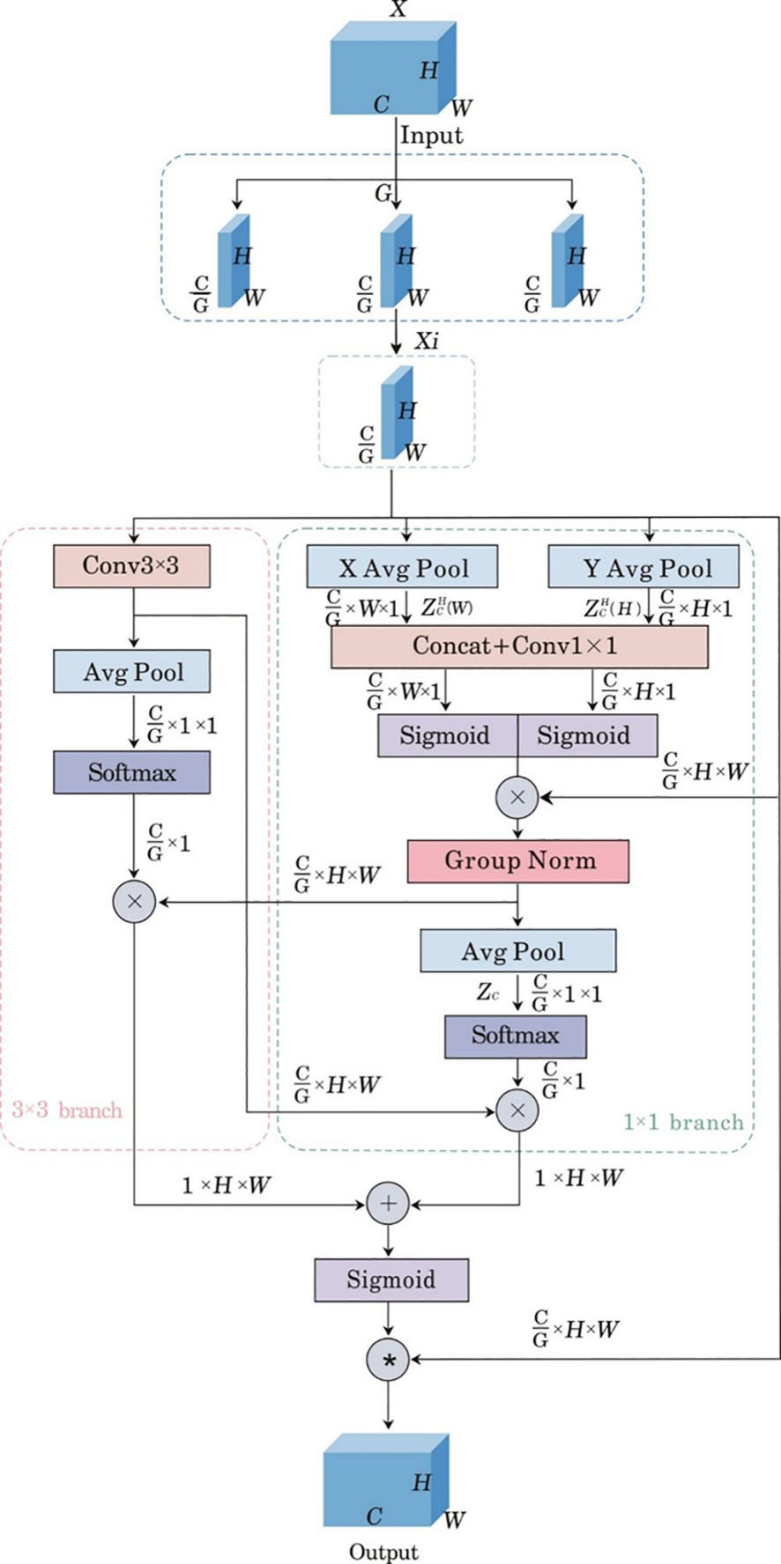
Network structure diagram of EMA attention mechanism. X denotes the input feature map, C, H and W represent the number of channels, height, and width of the input image, respectively, G represents the number of groups, C/G represents the number of channels contained in each group, and $Z_{C}$ represents the feature map of the c-th channel after two-dimensional global average pooling, $X_{i}$ represents the sub-feature map, “X Avg Pool” and “Y Avg Pool” denote one-dimensional average pooling operations along different directions, while “Avg Pool” refers to two-dimensional average pooling, “Group Norm” represents group normalization, Sigmoid refers to the activation function, Softmax denotes the normalization function.

In the task of pest and disease identification in pakchoi, challenges such as severe occlusion, complex background interference, and poor image quality often hinder the effective extraction of features from small targets. To address this issue, this paper introduces the Efficient Multi-scale Attention (EMA) mechanism. The EMA mechanism employs cross-spatial learning to group channels without reducing their dimensionality, thereby preserving information across each channel while minimizing computational overhead (Liu et al., 2024). The network structure of the EMA attention mechanism is illustrated in **Figure 5**.

When EMA operates, first, it takes the feature map $\text{X}\in {\text{R}}^{\text{C}\times \text{H}\times \text{W}}$ extracted by the backbone network as input, and partitions the feature map into G groups of sub-feature maps along the channel dimension of X: $\text{X}=\left[{\text{X}}_{0},{\text{X}}_{1},\cdots ,{\text{X}}_{\text{i}},\cdots ,{\text{X}}_{\text{G}-1}\right]$, where each sub-feature map ${\text{X}}_{\text{i}}\in {\text{R}}^{\frac{\text{C}}{\text{G}}\times \text{H}\times \text{W}}$. Subsequently, in the 1×1 branch, two one-dimensional global average pooling operations are performed along the horizontal and vertical axes to encode channels, establishing interactions between channel and spatial location information, while generating two spatial encoding feature maps that are concatenated along the vertical direction. This operation is computed as follows (Equations 1 and 2):

(1) $$\begin{array}{c} {\text{Z}}_{\text{C}}^{\text{H}}\left(\text{H}\right)=\frac{1}{\text{W}}\sum_{0\leq \text{i}\leq \text{W}}{\text{X}}_{\text{C}}\left(\text{H},\text{i}\right) \end{array}$$

(2) $$\begin{array}{c} {\text{Z}}_{\text{C}}^{\text{W}}\left(\text{W}\right)=\frac{1}{\text{W}}\sum_{0\leq \text{j}\leq \text{H}}{\text{X}}_{\text{C}}\left(j,\text{W}\right) \end{array}$$

In the formula, H and W are the height and width of the feature map, respectively; ${\text{Z}}_{\text{C}}^{\text{H}}\left(\text{H}\right)$ and ${\text{Z}}_{\text{C}}^{\text{W}}\left(\text{W}\right)$ are the axis-specific pooling outputs generated along the horizontal axis and vertical axis, respectively; i and j are the width and height of the input of the C-th channel, respectively; ${\text{X}}_{\text{C}}\left(\text{j},\text{W}\right)$ and ${\text{X}}_{\text{C}}\left(H,i\right)$ are the input features at the spatial positions (j, W) and (H, i) in the C-th channel, respectively.

Subsequently, a nonlinear Sigmoid activation function is adopted to aggregate the two spatial encoding feature maps processed by 1×1 convolution in each group. Then, through group normalization, 2D average pooling, and Softmax operation in sequence, an intermediate feature map with a dimension of C/G×1 is generated (Liu et al., 2024). The 2D global average pooling operation applied to the processed feature is described by Equation 3:

(3) $$\begin{array}{c} Z_{C}=\frac{1}{\text{H}\times \text{W}}\sum_{\text{j}}^{\text{H}}\sum_{\text{j}}^{\text{W}}{\text{X}}_{\text{C}}\left(\text{i},\text{j}\right) \end{array}$$

In the formula, $Z_{C}$ represents the feature map of the C-th channel after 2D global average pooling, and $X_{C}\left(i,j\right)$ represents the processed feature at the spatial position (i,j) in the C-th channel after 1×1 convolution and Sigmoid activation. The intermediate feature map after the Softmax operation is subjected to matrix multiplication with the sub-feature map processed by 3×3 convolution, resulting in the first spatial attention weight map with a dimension of 1×H×W.

The output from the 3×3 branch, after 2D average pooling and Softmax operation, undergoes matrix multiplication with the feature map from the 1×1 branch, generating the second spatial attention weight map with dimensions 1×H×W. Finally, the two spatial attention weight maps are summed and then normalized via the Sigmoid function to obtain the final attention weight map. This weight map is subsequently mapped with the original feature map, enabling the model to focus attention on key regions.

##### 2.5.1.2 Partial convolution module

**Figure 6:**
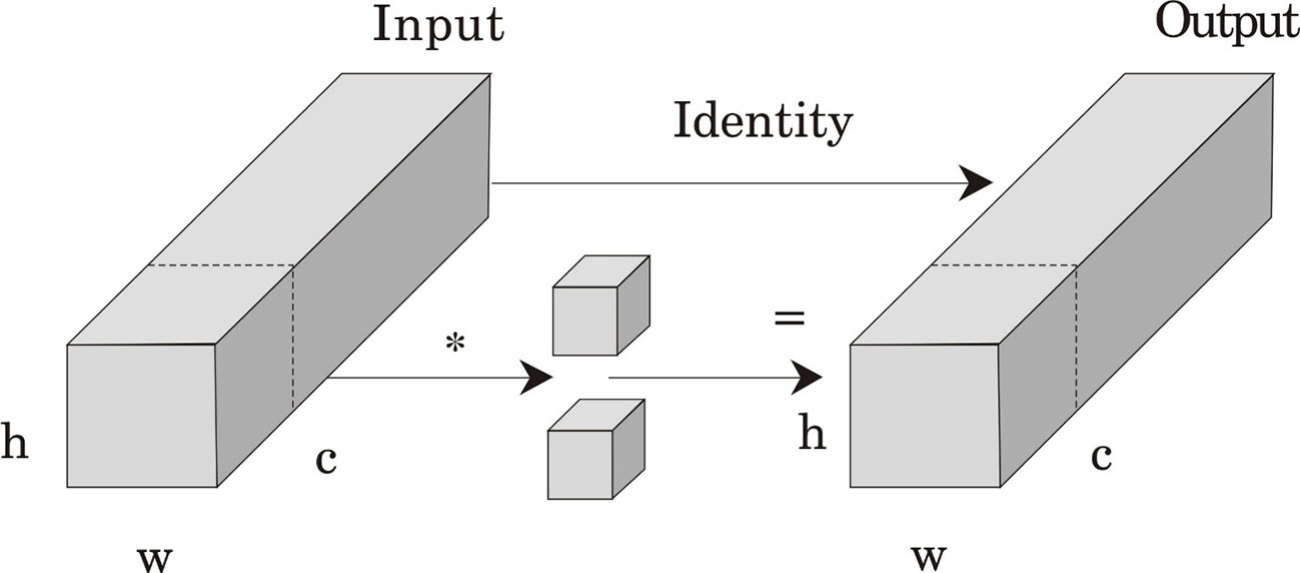
Structure diagram of the partial convolution module.

PConv is an efficient convolutional structure, which has the advantages of flexibility and adaptability to data loss compared to traditional convolutions. PConv does not always use the same convolution kernel for all input data, but dynamically determines the scope of the convolution kernel based on the validity of the data, that is, whether the data features are missing or damaged, and suppresses the interference of irrelevant factors. This design minimizes unnecessary computation and memory access, significantly improving the real-time processing efficiency of the model. It can reduce floating-point operations while maintaining high feature extraction capabilities, effectively processing images with irregular missing or occluded features (**Figure 6**).

##### 2.5.1.3 Fusion and structure of the C2f-PE module

**Figure 7:**
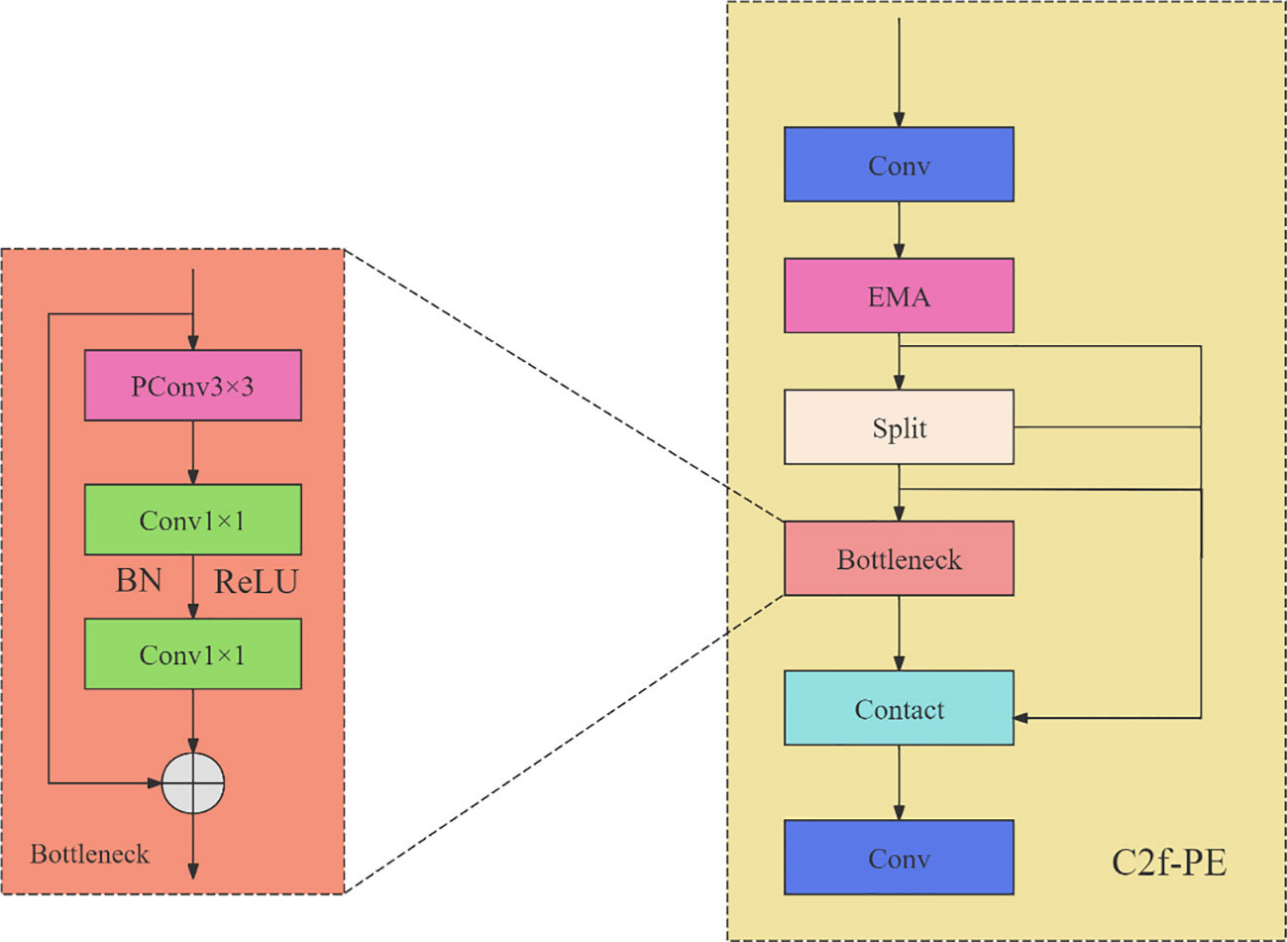
Structure diagram of C2f-PE module.

To enhance the model performance, this study integrates the EMA attention mechanism and PConv into the C2f module to construct a novel C2f-PE module (whose structure is shown in **Figure 7**). Specifically, the EMA attention mechanism is first inserted after the first Conv in the C2f module to dynamically allocate the weights of input features; meanwhile, the 3×3 standard convolution in the Bottleneck is replaced by 3×3 PConv for lightweighting. Based on pre-experiments, replacing the 4th C2f module in the backbone network with C2f-PE achieves the optimal effect. This replacement ensures the stability of the input and output dimensions of each layer in the network and ultimately enables the model to show stronger detection capability for pakchoi pest and disease images with irregular missing or leaf occlusion.

#### 2.5.2 Feature fusion network BiFPN

**Figure 8:**
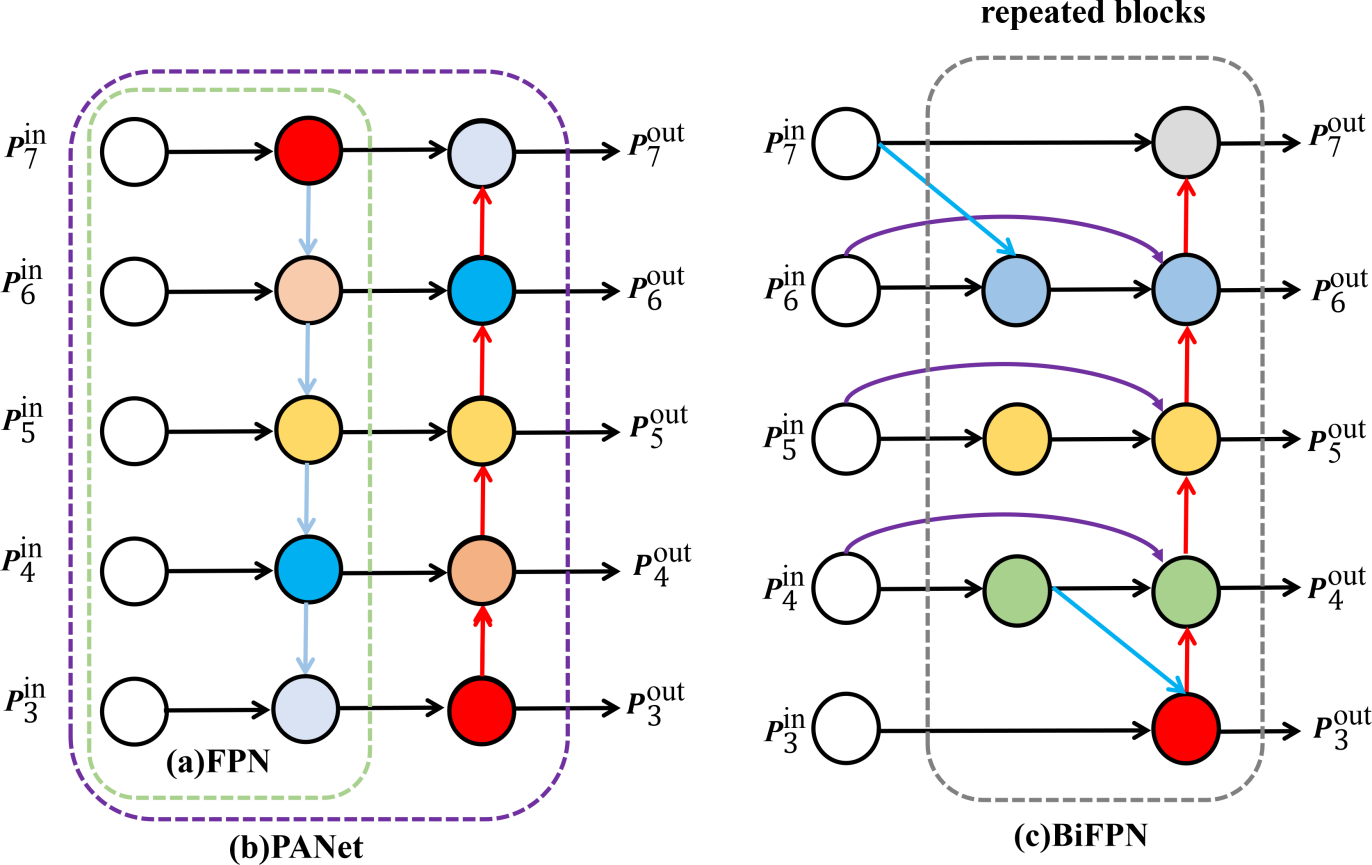
FPN, PANeT and BiFPN structure diagram.

YOLOv8n employs a feature pyramid structure composed of FPN (Feature Pyramid Network) and PAN (Path Aggregation Network) (Liu et al., 2018) to achieve cross-scale feature fusion: as shown in **Figure 8a**, the Feature Pyramid Network (FPN) transmits high-level semantic features from top to bottom. Conversely, the Path Aggregation Network (PAN) enhances low-level localization features through bottom-up paths, and the two jointly establish multi-scale feature correlations (**Figure 8b**). However, due to its feature aggregation mechanism, PANet has inherent limitations in pakchoi pest and disease detection. First, the multi-scale downsampling and fusion processes of PANet lead to the gradual attenuation and loss of detailed features layer by layer, resulting in the loss of integrity of small target features. Second, the feature fusion strategy of PANet has insufficient robustness to background noise and illumination interference, making it difficult to separate features of occluded targets and easily causing feature confusion and detection deviations. Third, the feature aggregation paths of PANet are relatively complex with large computational overhead, making it difficult to meet the requirements of real-time detection tasks. To address the above issues, this study introduces the Bidirectional Feature Pyramid Network (BiFPN) (Tan et al., 2020), which possesses bidirectional feature flow and a dynamic weight learning mechanism, as the core feature fusion module. Its advantages are mainly reflected in three aspects: First, structural optimization. The network prunes redundant nodes to reduce ineffective computations and adds cross-layer connections to enhance direct feature interaction. Second, dynamic weighting. It employs learnable weights with fast normalization to fuse features across different scales, adaptively focusing on highly discriminative regions. The weighted fusion is defined by Equation 4 as follows:

(4) $$\begin{array}{c} 0=\sum_{\text{i}}\frac{{\text{W}}_{\text{i}}}{\epsilon +\sum_{\text{j}}{\text{W}}_{\text{j}}}{\text{l}}_{\text{j}} \end{array}$$

In the formula: ${\text{W}}_{\text{i}}$ denotes the learnable weight. After calculating ${\text{W}}_{\text{i}}$, it is processed by a ReLU activation function to ensure ${\text{W}}_{\text{i}}\geq 0$. ϵ is a constant, usually set to 0.0001 to avoid numerical instability. ${\text{l}}_{\text{j}}$ denotes the input feature.

Third, efficiency enhancement. By simplifying computational pathways and reducing model complexity, BiFPN achieves a synergistic optimization of accuracy and inference speed. This study leverages its capability for precise weighted fusion of cross-scale features to enhance the retention of small target details and feature discriminability in complex backgrounds. Through accurate weighting, BiFPN preserves fine details of small objects and strengthens feature discrimination in dense/occluded scenarios, while its high efficiency readily adapts to the computational constraints of edge devices, making it an ideal solution for improving the accuracy and practical efficiency of pest and disease detection. The structure diagram of BiFPN is shown in **Figure 8**.

#### 2.5.3 Wise-IoU loss function

YOLOv8n employs CIoU (Zheng et al., 2020) as its bounding box regression loss function, whose calculation formula is shown in Equation 5:

(5) $$\begin{array}{c} {\text{L}}_{\text{C}\text{I}\text{o}\text{U}}=1-\text{I}\text{o}\text{U}+\frac{{\text{p}}^{2}\left(\text{b},{\text{b}}^{\text{g}\text{t}}\right)}{{\text{c}}^{2}}+\alpha \nu \end{array}$$

In the formula, α is the balanced weight coefficient; ν is a term for calculating the consistency of the aspect ratio between the predicted bounding box and the ground truth box; b and $b^{gt}$ are the center coordinates of the predicted bounding box and the ground truth box, respectively; c denotes the diagonal length of the minimum enclosing rectangle of the predicted bounding box and the ground truth box; $p^{2}$ denotes the distance between the center points of the predicted bounding box and the ground truth bounding box.

**Figure 9:**
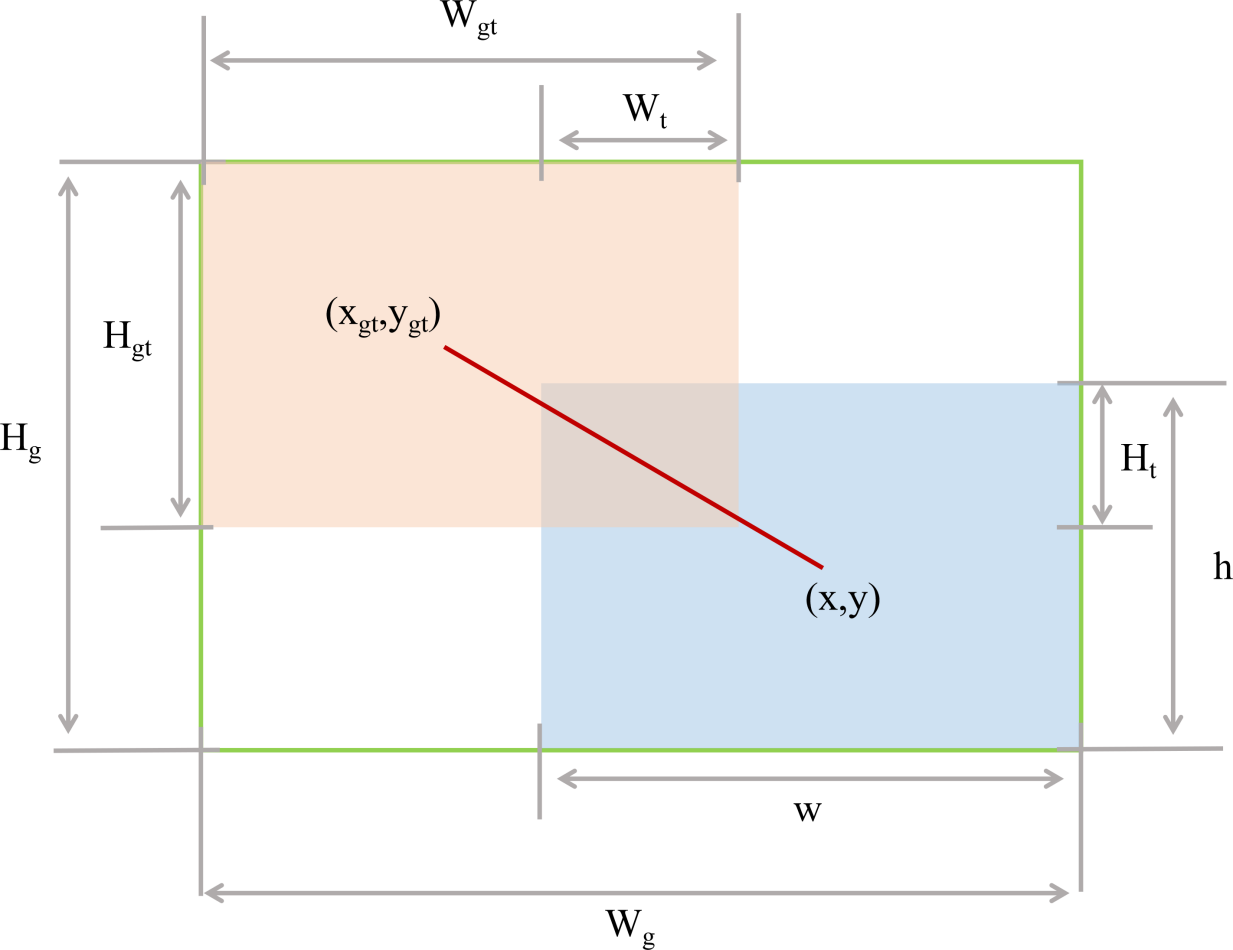
Wise-IoU loss function.

To specifically improve the model’s detection performance for small target pests and diseases, this study introduces the Wise-IoU (WIoU) loss function based on a dynamic non-monotonic focusing mechanism to balance samples. This strategy not only reduces the competitiveness of high-quality anchor boxes but also mitigates the harmful gradients generated by low-quality examples. This enables WIoU to focus on anchor boxes of average quality and improve the overall performance of the detector, as shown in **Figure 9**. ${\text{W}}_{\text{t}}$ and ${\text{H}}_{\text{t}}$ denotes the width and height of the overlapping region between the ground-truth bounding box and the predicted bounding box; (x, y) denotes the center coordinates of the predicted bounding box; ( ${\text{x}}_{\mathrm{gt}}$, $y_{gt}$) denotes the center coordinates of the ground truth box; w and h indicate the width and height of the prediction box; ${\text{W}}_{\mathrm{gt}}$ and ${\text{H}}_{\mathrm{gt}}$ indicate the width of the target box; ${\text{W}}_{\text{g}}$ and $H_{g}$ indicates the minimum border width and height.

Since training data inevitably contain low-quality examples, IoU is replaced with outlier degree through the dynamic non-monotonic focusing mechanism to evaluate the quality of anchor boxes, so as to avoid excessive penalties on the model caused by geometric factors (e.g., distance and aspect ratio), as shown in Equations 6-8.

(6) $$\begin{array}{c} {\text{L}}_{\text{W}\text{I}\text{o}\text{U}}=\text{r}\cdot {\text{R}}_{\text{W}\text{I}\text{o}\text{U}}{\text{L}}_{\text{I}\text{o}\text{U}},\text{r}=\frac{\text{β}}{\text{δ}{\text{α}}^{\text{β}-\text{α}}} \end{array}$$

(7) $$\begin{array}{c} \text{β}=\frac{{\text{L}}_{\text{I}\text{o}\text{U}}^{*}}{\bar{{\text{L}}_{\text{I}\text{o}\text{U}}}}\in [0,+\infty ) \end{array}$$

(8) $$\begin{array}{c} {\text{R}}_{\text{W}\text{I}\text{o}\text{U}}=\text{e}\text{x}\text{p}\left(\frac{{\left(\text{x}-{\text{x}}_{\text{g}\text{t}}\right)}^{2}+{\left(\text{y}-{\text{y}}_{\text{g}\text{t}}\right)}^{2}}{{\left({\text{c}}_{\text{w}}^{2}+{\text{c}}_{\text{h}}^{2}\right)}^{*}}\right) \end{array}$$

In the formula, ${\text{L}}_{\mathrm{IoU}}\in \left[0,1\right]$ denotes the IoU loss, which will weaken the penalty term for high-quality anchor boxes and strengthen its focus on the distance between center points when the overlap between the anchor box and the predicted bounding box is high; ${\text{R}}_{\mathrm{WIoU}}\in \left[1,\exp\right]$ denotes the penalty term of Wise-IoU, which strengthens the loss of anchor boxes of average quality. Superscript * denotes that it does not participate in backpropagation, which effectively prevents the network model from generating non-convergent gradients. $\bar{{\text{L}}_{\mathrm{IoU}}}$ serves as a normalization factor, denoting the incremental moving average. β denotes the outlier degree: the smaller its value, the higher the anchor box quality; the larger its value, the lower the anchor box quality. Based on this, a bidirectional gradient gain adjustment strategy is designed: for high-quality anchor boxes with low β, small gradient gains are assigned; for weak-feature anchor boxes with high β, large gradient gains are assigned. This effectively reduces harmful gradients from low-quality training samples, so as to make the bounding box regression loss focus on anchor boxes of average quality, and ultimately improve the detection robustness of the network to pakchoi pest and disease scenarios.

### 2.6 Model training and evaluation metrics

#### 2.6.1 Experimental environment and training strategies

The experiments were conducted on a Windows 11 system, with the deep learning model implemented in PyTorch. Experimental environment parameters are summarized in **Table 3**.

**Table 3:** Training environment and hardware platform parameters table.

Parameters	Configuration
CPU	AMD Ryzen7-7735H
GPU	NVIDIA GeForce RTX 4090
GPU memory size	24GB
Operating systems	Windows 11
Deep learning architecture	PyTorch 2.3.1 + Python3.9.19 + CUDA12.1

The hyperparameters were configured to optimize model training and validation efficiency while maintaining performance and accuracy. Detailed settings are listed in **Table 4**.

**Table 4:** Some key parameters set during model training.

Parameters	Setup
Epochs	200
Batch size	32
Input image size	640×640
Workers	8
Initial learning rate	0.01
Optimizer	SGD
Weight decay	0.0005
Momentum	0.937
Close mosaic	10
Patience	50

#### 2.6.2 Model evaluation indicators

To comprehensively evaluate the performance of the multi-scenario small target detection model for pakchoi pests and diseases, this study adopts Precision (P), Recall (R), mean average precision (mAP), Floating-point Operations (FLOPs), Parameters, and model size (MB) as evaluation metrics. Based on the matching relationship between ground truth annotations and prediction results in object detection tasks, samples are classified into four categories: True Positives (TP, predicted as positive and actually positive), False Positives (FP, predicted as positive but actually negative), True Negatives (TN, predicted as negative and actually negative), and False Negatives (FN, predicted as negative but actually positive). The calculations of relevant metrics are shown in Equations 9-12.

(9) $$\begin{array}{c} \text{P}=\frac{{\text{T}}_{\text{p}}}{{\text{T}}_{\text{p}}+{\text{F}}_{\text{N}}}\times 100\% \end{array}$$

(10) $$\begin{array}{c} \text{R}=\frac{{\text{T}}_{\text{P}}}{{\text{T}}_{\text{P}}+{\text{F}}_{\text{N}}}\times 100\% \end{array}$$

(11) $$\begin{array}{c} \text{A}\text{P}=\int_{0}^{1}\text{P}\left(\text{R}\right)\text{d}\text{R} \end{array}$$

(12) $$\begin{array}{c} \mathrm{mAP}=\frac{1}{n}\sum_{i=1}^{n}AP_{i}mAP=\frac{1}{n}\sum_{i=1}^{n}AP_{i} \end{array}$$

Herein, Precision (P) reflects the reliability of predicted positive samples; Recall (R) reflects the model’s ability to identify true positive samples. Average Precision (AP) denotes the average precision of a specific category, while mean Average Precision (mAP) represents the average of the average precisions across all categories. The larger the mAP value, the higher the average precision of the model and the better the detection performance.

## 3 Results

### 3.1 Analysis of convergence experiment

Visualization of loss curves can intuitively reflect the convergence process of the model, thereby facilitating better adjustment of training strategies. The loss values include bounding box loss and distribution focal loss (used to evaluate the regression performance of object detection bounding boxes) as well as classification loss (used to evaluate classification performance) (Yang et al., 2023).

To systematically evaluate the stability of model performance and mitigate interference from random factors during training, this study conducted independent and repeated training and evaluation experiments for each model. The specific procedures were as follows: All experiments were performed under identical hardware (e.g., GPU model, memory configuration) and software (e.g., deep learning framework version, operating system) environments. Each model was trained independently for five repeated runs. Before each run, the model parameters and weights were reinitialized, and the input order of the training dataset was randomly shuffled to eliminate the influence of initial weights and data sequence on the results. The final reported performance metrics (e.g., accuracy, mAP) were calculated as the arithmetic mean of the results from the five runs, serving to quantify the stability of model performance. All model comparisons were based on these averaged metrics to ensure fairness and reliability in the evaluation.

**Figure 10:**
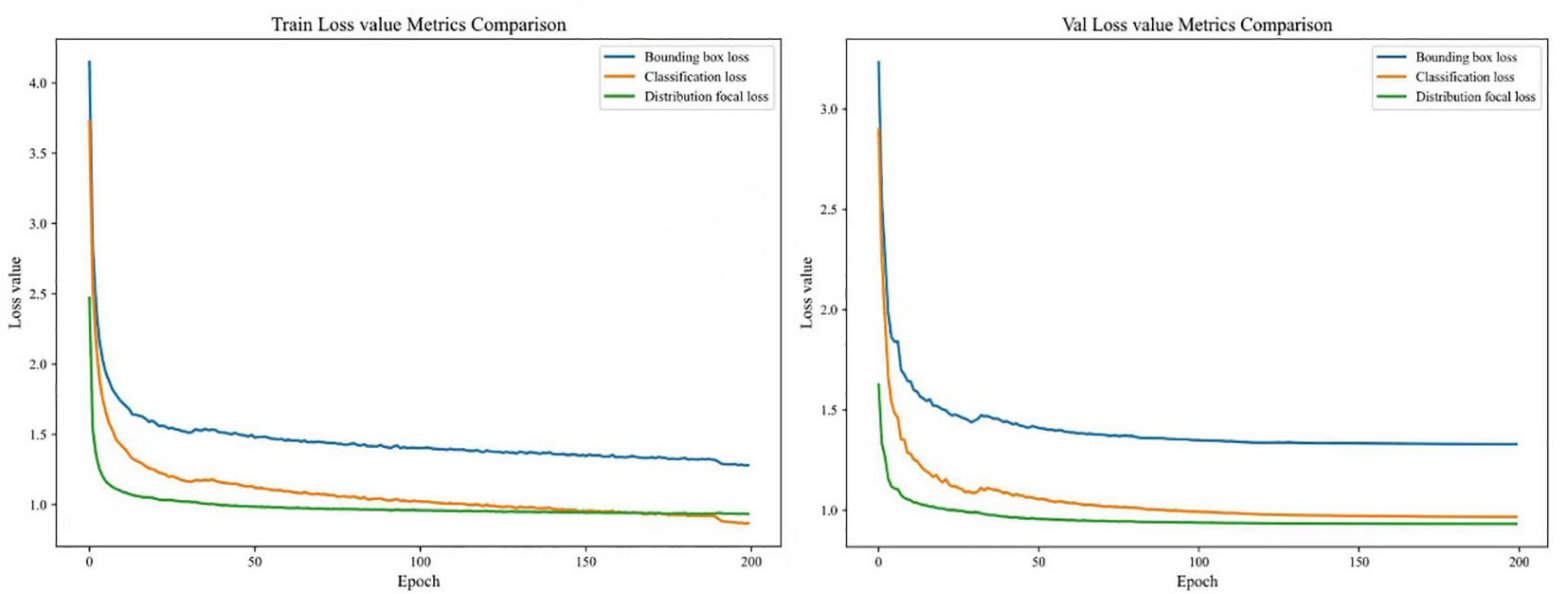
Loss value curves of YOLOv8-DBW.

As shown in **Figure 10**, throughout the training process, the model exhibited no signs of overfitting or underfitting, indicating that it possesses good generalization ability and the capability to capture data patterns. As the number of training epochs increased, all three types of loss values decreased continuously. After the 130th epoch, the loss curves tended to converge and stabilize, suggesting that the model had reached the optimal state and could proceed to the stage of model performance evaluation.

### 3.2 Comparison of different attention mechanisms

To verify the rationality of introducing the EMA attention mechanism, this study independently embedded it into the backbone network of the original YOLOv8n model. Meanwhile, the four attention mechanisms (SE, CA, ECA, and CBAM) were respectively introduced at the same position to conduct comparative experiments, and the experimental results are shown in **Table 5**. Data indicate that the model with the EMA attention mechanism introduced achieves an Accuracy of 83.2% and a mean average precision (mAP) of 80.1%, with both indicators being higher than those of the models incorporating the other four attention mechanisms. In addition, the Recall of this model is 71.6%, which is only 0.4 percentage points lower than that of the model with the SE attention mechanism introduced. Overall, the EMA mechanism shows obvious advantages in terms of core detection accuracy indicators.

**Table 5:** Comparison of the effects between different attention mechanisms.

Attention mechanisms	P/%	R/%	mAP/%
EMA	83.2	71.6	80.1
SE	82.0	72	79.5
CA	83.0	70.6	79.8
ECA	83.1	70.4	79.9
CBAM	82.9	68.8	79.7

### 3.3 Ablation experiment

To verify the effectiveness of each improvement in the modified YOLOv8n, this study set up 8 ablation experiment schemes to validate the effectiveness of the modified YOLOv8n modules, with the results shown in **Table 6**.

**Table 6:** Results of ablation experiment.

Test	C2f-PE	BiFPN	Wise-IoU	P/%	R/%	mAP/%	FLOPs/G	Parameters/M	Model size/MB
1	–	–	–	81.4	69.8	77.8	8.1	3.0	6.3
2	✓	–	–	83.3	70.8	80.5	7.3	2.7	5.8
3	✓	✓	–	85.7	74.1	84.3	7.0	2.0	4.3
4	✓	✓	✓	86.4	75.3	85.3	7.0	2.0	4.3
5	–	✓	–	82.4	71.5	78.8	7.1	1.9	4.2
6	–	✓	✓	83.2	73.5	81.5	7.1	1.9	4.2
7	–	–	✓	82.9	71.0	79.3	8.1	3.0	6.3
8	✓	–	✓	84.3	71.6	82.5	7.3	2.7	5.8

P denotes precision, R denotes recall, and mAP represents the mean average precision. “√” indicates the use of this improvement, while “-” signifies that the improvement was not applied.

As shown in **Table 6**, after introducing the C2f-PE module, the feature extraction capability of the model was notably enhanced compared with the original baseline. Specifically, Precision (P), Recall (R), and mean Average Precision (mAP) increased by 1.9, 1.0, and 2.7 percentage points, respectively. This improvement can be attributed to the EMA attention mechanism, which effectively suppresses interference caused by occlusion and enhances the model’s focus on small-target features. Meanwhile, due to the lightweight convolutional design of the PConv module, the number of floating-point operations (FLOPs) was reduced by 0.8 G, while the model size and parameter count decreased by 0.3 MB and 0.5 M, respectively. After further integrating the BiFPN module, the model achieved additional performance gains and improved computational efficiency. Compared with the original model, P, R, and mAP increased by 4.3, 4.3, and 6.5 percentage points, respectively. At the same time, the number of parameters and model size were reduced by 33.3% and 31.8%, while FLOPs decreased by 13.6%, indicating a significant improvement in lightweight performance. These results demonstrate that BiFPN effectively enhances multi-scale feature fusion while reducing redundant computations. Finally, after replacing the original loss function with the Wise-IoU loss, the model’s detection performance was further improved, with P, R, and mAP increasing by 5.0, 5.5, and 7.5 percentage points, respectively. This result suggests that Wise-IoU improves the accuracy and stability of bounding box regression, thereby enhancing overall detection robustness. Based on the ablation experiment results, each individual module contributes positively to performance improvement. When all proposed modules are combined, the model achieves optimal performance across all evaluation metrics, confirming the effectiveness of the proposed improvements.

### 3.4 Analysis of comparative experiments on different IoU losses

To verify the effectiveness of the proposed Wise-IoU loss function in the pakchoi pest and disease detection task, training was conducted using YOLOv8’s default CIoU as well as existing mainstream regression loss functions including DIoU, GIoU, SIoU, and EIoU. The evaluation metrics adopted mAP at IoU thresholds of 0.5 and 0.5–0.95. The experimental results are shown in **Table 7**. As indicated in **Table 6**, compared with the default CIoU of YOLOv8n, the proposed Wise-IoU increased mAP@0.5 and mAP@0.5:0.95 by 1.5 and 1.3 percentage points respectively. Among all comparative methods, Wise-IoU achieved the optimal precision, verifying its superiority in agricultural disease detection scenarios.

**Table 7:** Performance comparison of different IoU loss.

Model	mAP@0.5	mAP@0.5:0.95
YOLOv8n + CIoU	77.8	59.5
YOLOv8n + DIoU	76.2	58.2
YOLOv8n + GIoU	77.6	59.1
YOLOv8n + SIoU	77.5	59.2
YOLOv8n + EIoU	77.4	59.7
YOLOv8n + Wise-IoU	79.3	60.8

mAP@0.5 and mAP@0.5:0.95 represent the mean average precision at IoUthresholds of 0.5 and 0.5 to 0.95.

### 3.5 Comparison of mainstream object detection models

To evaluate the performance of the proposed YOLOv8-DBW model, comparative experiments were conducted against several mainstream object detection methods, including Faster R-CNN, SSD, YOLOv5s, YOLOv5n, YOLOv7-tiny, YOLOv10n, YOLOv11n, and YOLOv12n. To ensure fairness and scientific rigor, all benchmark models were retrained on the same pakchoi pest and disease dataset using identical hardware environments and hyperparameter configurations, as specified in **Tables 3** and **4**. To minimize the effects of experimental randomness, each model was independently trained five times, and the reported performance metrics represent the arithmetic mean of the five runs. The comparison results are summarized in **Table 8**. As shown in **Table 8**, the proposed YOLOv8-DBW model achieved superior detection performance compared with all benchmark models. Specifically, its mean average precision (mAP) exceeded that of Faster R-CNN, SSD, YOLOv5s, YOLOv5n, YOLOv7-tiny, YOLOv10n, YOLOv11n, and YOLOv12n by 23.4, 19.1, 10.0, 11.5, 14.0, 6.9, 7.8, and 12.1 percentage points, respectively. Meanwhile, the number of model parameters was reduced by 96.7%, 94.4%, 71.5%, 20.0%, 66.7%, 25.9%, 20.0%, and 20.0% compared with the corresponding models. Although the FLOPs of YOLOv8-DBW are slightly higher than those of YOLOv5n and approximately 0.6 G and 1.0 G higher than those of YOLOv11n and YOLOv12n, respectively, they remain substantially lower than those of the other compared models. In addition, the model size of YOLOv8-DBW is reduced by 96.7%, 91.3%, 73.7%, 5.3%, 69.5%, 33.3%, 30.8%, and 29.5%, respectively, meeting the requirements for lightweight deployment. Although YOLOv5n, YOLOv11n, and YOLOv12n achieve higher inference speeds in terms of frames per second (FPS), their parameter counts and model sizes are larger than those of YOLOv8-DBW, and their detection accuracy remains lower. Overall, the proposed YOLOv8-DBW model demonstrates a more favorable balance among detection accuracy, computational efficiency, and model compactness.

**Table 8:** Performance comparison of mainstream models.

Models	Precision/%	Recall/%	mAP@0.5/%	FLOPs/G	Parameters/M	FPS	Model size/MB
Faster-RCNN	63.8	62.1	61.9	121.4	60.1	38.5	108.6
SSD	65.3	60.2	66.2	61.2	35.2	41.7	41.3
YOLOv5s	79.1	70.7	75.3	15.7	7.0	69.8	13.7
YOLOv5n	75.3	71.3	73.8	4.2	2.5	99.3	3.8
YOLOv7-tiny	71.2	69.5	71.3	13.2	6.0	78.8	11.8
YOLOv10n	84.4	68.8	78.4	8.4	2.7	88.6	5.4
YOLOv11n	75.9	74.7	77.5	6.4	2.5	97.4	5.2
YOLOv12n	78.2	70.7	73.2	6.0	2.5	111.2	5.1
YOLOv8n-DBW	86.4	75.3	85.3	7.0	2.0	95.1	4.3

**Figure 11:**
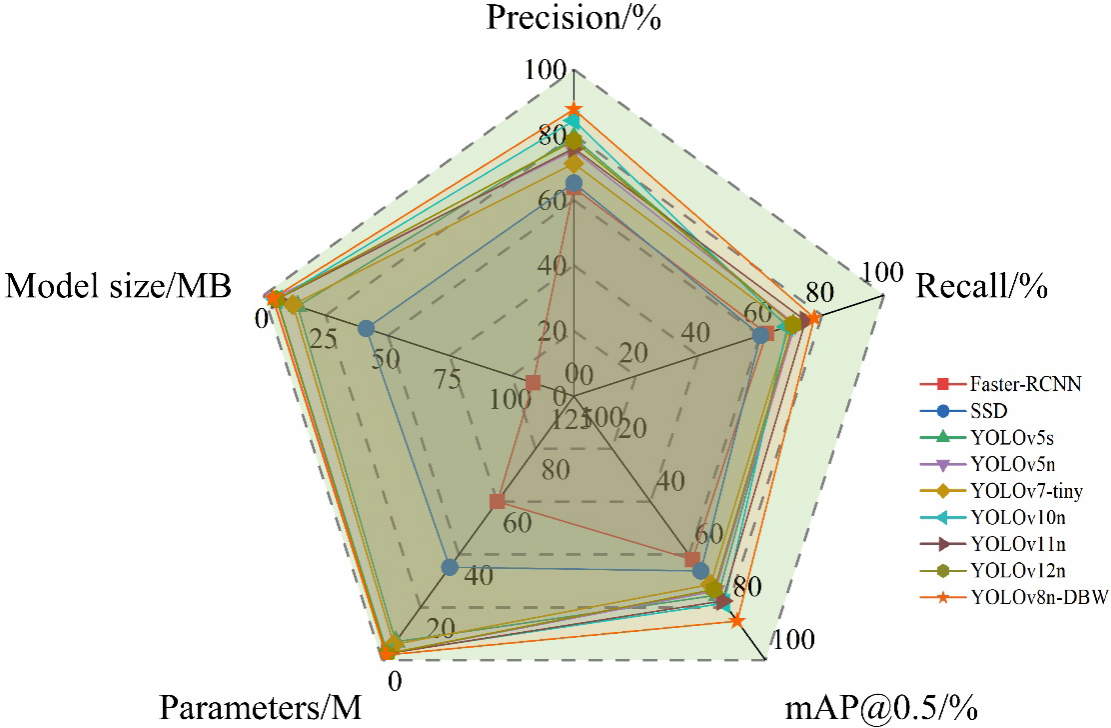
Radar chart of the comprehensive performance comparison of the mainstream.

The radar chart results characterizing the comprehensive performance of the models (**Figure 11**) show that the improved YOLOv8-DBW model has the most full and complete area, indicating that its performance in all aspects is closer to the ideal state compared with other models. In summary, the YOLOv8-DBW algorithm proposed in this study has demonstrated its superiority in multiple metrics.

### 3.6 Performance across different categories

To further evaluate the robustness of the proposed model under class imbalance, detailed performance metrics for each of the seven pest and disease categories are summarized in **Table 9**. Despite the variation in sample counts among different categories, the YOLOv8-DBW model achieved consistently strong performance across all classes. Specifically, the minority class Black Rot attained an mAP of 83.9%, which is highly comparable to that of the majority class Diamondback Moth at 87.4%. This relatively balanced performance across categories suggests that the proposed model is less sensitive to sample imbalance. Such robustness can be attributed to the synergistic effect of the enhanced feature extraction capability provided by the C2f-PE module and the dynamic sample weighting mechanism introduced by the Wise-IoU loss function, which together help mitigate potential bias toward majority classes.

**Table 9:** Detailed recognition performance for the seven categories.

Category	Sample count	Precision (%)	Recall (%)	mAP@0.5 (%)
Diamondback Moth	1085	88.5	78.2	87.4
Leaf Miner	842	87.1	77.0	86.2
Downy Mildew	992	85.9	74.5	84.8
Alternaria Leaf Spot	753	84.8	73.6	84.1
Black Rot	745	85.2	72.8	83.9
White Rust	855	86.5	75.4	85.5
White Spot	838	86.8	75.6	85.2

### 3.7 Model visualization analysis

**Figure 12:**
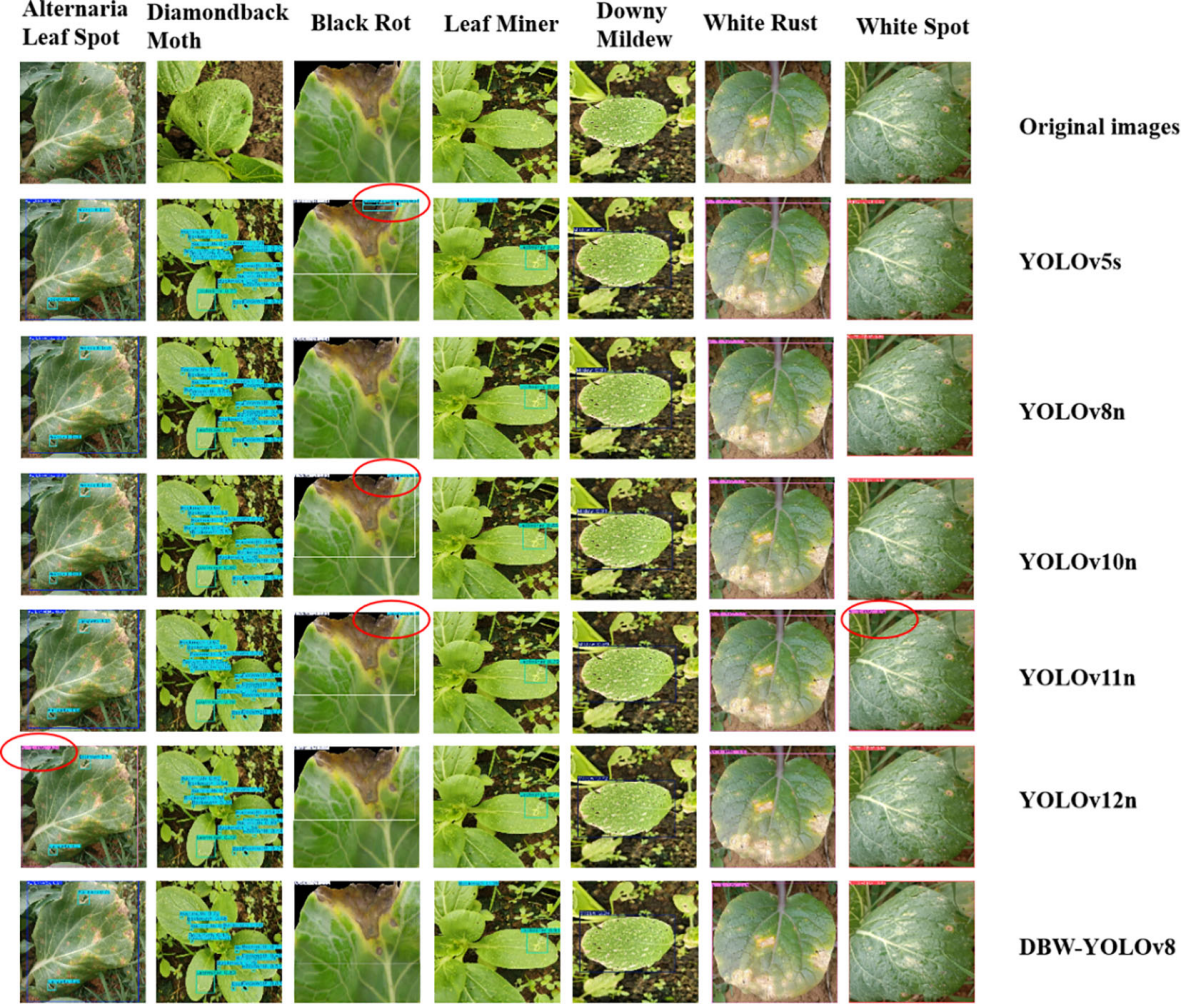
Recognition performance of different models for pakchoi diseases and pests. In the figure, red circles indicate false detections.

Based on the experimental results of mainstream models, by selecting and verifying better model algorithms for comparative analysis, the YOLOv5n, YOLOv10n, YOLOv11n, YOLOv12n, and the improved YOLOv8-DBW algorithm proposed in this study were chosen for visual comparison in the detection results of pakchoi pests and diseases, with the detection results shown in **Figure 12**.

As shown in **Figure 12**, the comparison of detection confidence on the test set reveals that some models exhibit noticeable cross-misclassification and background false detection issues in the identification of pakchoi pests and diseases. Specifically, YOLOv12 misidentifies Alternaria Leaf Spot as White Rust, and YOLOv11n makes a similar error in detecting White Spot. Moreover, in the detection of Black Rot, YOLOv5s, YOLOv10n, and YOLOv11n all misclassify background areas as Diamondback Moth. In contrast, the YOLOv8-DBW model shows no misclassification across all cases and achieves significantly higher detection accuracy than the other models. These results confirm that the improvements made to YOLOv8n effectively enhance the detection performance for pakchoi pests and diseases, addressing the insufficient accuracy of existing models.

## 4 Discussion

The experimental results indicate that the proposed YOLOv8-DBW model achieves superior performance compared with traditional object detection frameworks. Rather than relying on isolated improvements, the proposed architecture forms a synergistic design in which the C2f-PE module stabilizes feature representation, the BiFPN network enhances multi-scale feature fusion efficiency, and the Wise-IoU loss refines bounding box regression accuracy. This coordinated design effectively addresses the limitations of the original YOLOv8n model, particularly its insufficient detection accuracy for small and occluded pest and disease targets in complex field environments. With the rapid development of deep learning technologies (Mu and Zeng, 2019; Liu, 2022), their applications have become widespread, leading to significant breakthroughs in crop pest and disease recognition in recent years (Ai et al., 2020; Xin and Wang, 2021). Traditional recognition methods heavily rely on manual detection, which is not only time-consuming and labor-intensive but also prone to reducing efficiency and accuracy due to human errors. While existing approaches based on models such as CNN and YOLO have partially alleviated these issues (Zhai et al., 2020; Ma and Pang, 2023; Zhao and Liu, 2024), they still face obvious bottlenecks in computational efficiency and deployment on edge devices. In resource-constrained environments, high computational and storage demands often hinder practical application. For instance, an improved convolutional neural network (CNN) was used to construct a lightweight model for identifying common pests and diseases in winter wheat, achieving a recognition accuracy of 96.02% (Yao et al., 2023). A deep learning model trained for cassava disease detection achieved an accuracy of up to 98% (Ramcharan et al., 2017). Similarly, for jute plant diseases, a deep learning network named YOLO-JD was proposed, which achieved the best detection performance with a mean average precision (mAP) of 96.63% (Li et al., 2022). Therefore, optimizing the model structure to improve inference efficiency is key to enhancing its adaptability.

Compared to other vegetables, leafy vegetables such as pakchoi are more susceptible to pests and diseases due to their edible parts being close to the soil, weak stress resistance, and high environmental sensitivity. In recent years, factors such as abnormal climate, continuous cropping obstacles, and soil degradation have further increased the pressure on pest and disease control. The YOLO model offers prominent advantages for pakchoi pest and disease detection, including high precision, real-time performance, and quantifiability, forming the foundation for precision agriculture. He et al. (2025) proposed the FV-YOLOv5s model, which broke through the bottleneck of detecting weak features of two types of pests and diseases (diamondback moth and downy mildew) in leafy vegetables. Qiang and Shi (2022) addressed the problems of scattered small targets and missed detection of clusters in pakchoi pest detection under wide scenarios, constructing a technical chain of “block detection-hybrid model-edge-cloud collaboration” to realize accurate identification and efficient deployment of pests in wide scenarios. Zheng et al. (2024) focused on the issue of missed detection of small targets for two types of pakchoi pests, proposing the YOLOPC model based on the YOLOv5s model. By optimizing the network with the CBAM attention mechanism and dilated convolution, synergistic optimization of accuracy and lightweight performance were achieved. Recent studies have explored YOLO-based improvements for specific pest or disease categories; however, most existing approaches focus on single or limited target types. In contrast, the present study targets multi-category pakchoi pest and disease detection by integrating data augmentation strategies and a lightweight yet robust detection framework, enabling stable performance across diverse categories and field conditions.

The proposed YOLOv8-DBW model not only improves detection accuracy but also significantly reduces computational cost and model size, making it suitable for deployment on embedded and mobile devices. This balance between accuracy and efficiency provides practical technical support for real-time field monitoring and precision agriculture applications. Despite its strong performance, this study has several limitations. First, although the current dataset encompasses diverse environmental conditions across three provinces, further expansion to include a wider array of crop cultivars and distinct climatic zones would further bolster the model’s cross-region generalization ability. Second, the current model focuses on qualitative detection and does not provide quantitative assessment of disease severity. Third, the inherent black-box nature of deep learning models limits interpretability in agricultural decision-making scenarios. Future research should expand dataset diversity, integrate severity estimation methods, and incorporate interpretability techniques such as Grad-CAM to enhance model transparency and decision support capability. Overall, this study clarifies the direction for subsequent optimization and supports the transition from pest and disease detection toward precision decision support in agricultural production.

## 5 Conclusion

To achieve rapid and accurate intelligent detection of pakchoi pests and diseases, the present study proposes an online detection method named YOLOv8-DBW, based on an improved YOLOv8n architecture. The model incorporates three key enhancements. First, in the backbone network, the original C2f module is replaced with a proposed C2f-PE module that integrates Partial Convolution (PConv) and an Efficient Multi-scale Attention (EMA) mechanism, enhancing the model’s feature extraction capability, increasing its precision, recall, and mean average precision (mAP) by 1.9%, 1%, and 2.7%, respectively, while also achieving a preliminary level of lightweight design by reducing floating-point operations (FLOPs) by 0.8 G, model size by 0.3 MB, and the number of parameters by 0.5 M. Second, the BiFPN module is introduced to replace the original neck structure, which strengthens the model’s ability to detect overlapping or dense pest and disease instances under complex backgrounds. This modification leads to increases in precision, recall, and mAP of 4.3%, 4.3%, and 6.5%, respectively, while reducing parameters by 33.3%, model size by 31.8%, and FLOPs by 13.6%, significantly improving computational efficiency. Third, the Wise-IoU is adopted as the new bounding-box regression loss function, which improves the model’s ability to localize pest and disease features accurately, resulting in notable improvements in precision, recall, and mAP of 5.0%, 5.5%, and 7.5%, respectively. In the field of pakchoi pest and disease detection, the YOLOv8-DBW model shows significant improvements in the number of parameters, detection speed, and accuracy compared with classical object detection algorithms such as Faster R-CNN and SSD, as well as mainstream lightweight models including YOLOv5s, YOLOv5n, YOLOv7-tiny, YOLOv10n, YOLOv11n, and YOLOv12n. Therefore, for field cultivation, this model can be deployed on devices to identify pakchoi pests and diseases and provide early warning, and will also facilitate precision variable spraying of pesticides, thereby realizing precision and efficient prevention and control of pests and diseases.

## Data Availability

The original contributions presented in the study are included in the article/supplementary material. Further inquiries can be directed to the corresponding author.
